# Green banana resistant starch: A promising potential as functional ingredient against certain maladies

**DOI:** 10.1002/fsn3.4063

**Published:** 2024-05-06

**Authors:** Haroon Munir, Hamza Alam, Muhammad Tahir Nadeem, Riyadh S. Almalki, Muhammad Sajid Arshad, Hafiz Ansar Rasul Suleria

**Affiliations:** ^1^ Department of Food Science, Faculty of Life Sciences Government College University Faisalabad Faisalabad Pakistan; ^2^ Department of Pharmacology and Toxicology, Faculty of Pharmacy Umm AL‐Qura University Makkah Saudi Arabia; ^3^ Department of Agriculture and Food Systems The University of Melbourne Melbourne Victoria Australia

**Keywords:** functional properties, GBRS (green banana resistant), health perspectives, resistant starch, starch, starch application

## Abstract

This review covers the significance of green banana resistant starch (RS), a substantial polysaccharide. The food industry has taken an interest in green banana flour due to its 30% availability of resistant starch and its approximately 70% starch content on a dry basis, making its use suitable for food formulations where starch serves as the base. A variety of processing techniques, such as heat‐moisture, autoclaving, microwaving, high hydrostatic pressure, extrusion, ultrasound, acid hydrolysis, and enzymatic debranching treatments, have made significant advancements in the preparation of resistant starch. These advancements aim to change the structure, techno‐functionality, and subsequently the physiological functions of the resistant starch. Green bananas make up the highest RS as compared to other foods and cereals. Many food processing industries and cuisines now have a positive awareness due to the functional characteristics of green bananas, such as their pasting, thermal, gelatinization, foaming, and textural characteristics. It is also found useful for controlling the rates of cancer, obesity, and diabetic disorders. Moreover, the use of GBRS as prebiotics and probiotics might be significantly proved good for gut health. This study aimed at the awareness of the composition, extraction and application of the green banana resistant starch in the future food products.

## Graphical abstract

Resistant starch (RS), a significant polysaccharide, is one of the intriguing bioactive components of starch. Among cereals and other foods, green bananas make up the most RS. Green banana resistant starch (GBRS) has potential value in the future as a functional ingredient and a fat replacement.

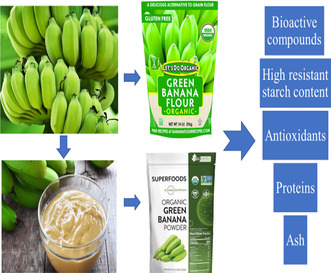

## 1 INTRODUCTION

**FIGURE 1:**
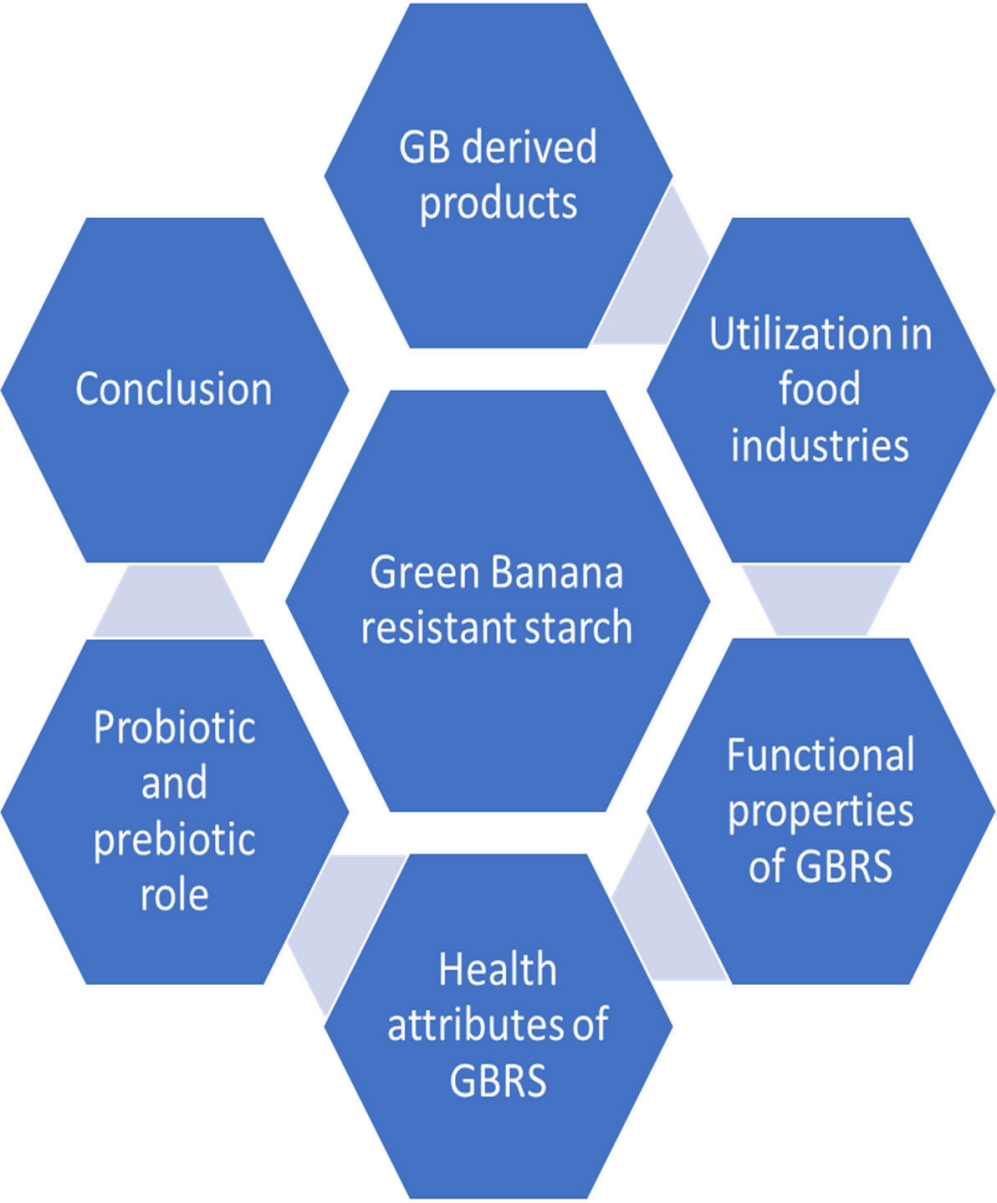
Cyclic view of topics.

Starch is absolutely necessary for the maintenance of a healthy diet in humans since it provides energy. Resistant starch is one of the supplements for fiber because of its prebiotic properties (Das et al., 2022a). The component of a food known as “resistant starch” is the fraction that is not engaged by the small intestine but is instead fermented by bacteria in the colon (Vaidya & Sheth, 2011). Starches can be classified according to their behavior when incubated with enzymes. Resistant starch was determined by subtracting the rapidly digestible starch (RDS)and slowly digestible starch (SDS) from the total starch (TS). RS is further classified as RS1, RS2, RS3, RS4, and RS5 based on in real life digestion and related to physiological properties (Gutiérrez & Tovar, 2021; Sajilata et al., 2006). Because of its nutritional composition and possible health advantages, green banana products are gaining popularity. Nutritionally, the unripe or green banana has a lot going for it. It has plenty of good stuff including bioactive compounds, minerals, and vitamins. Green banana flour is a good and healthier unconventional to wheat flour for use in bread and pasta due to its 74% Resistant Starch content (Burkhart et al., 2022). Consuming unripe bananas may benefit human health, according to some research. This is based on the notion that unripe bananas have the highest concentration of RS of any unprocessed food. Bananas are an outstanding source of fiber, vitamins, minerals, and resistant starch that may all have health benefits, especially when they are still green (Chuathong et al., 2021). Polyphenol, antioxidant, peptides, and phytosterols are abundant in unripe green banana powder (Bennett et al., 2010; Oliveira et al., 2008). Because of their high quantities of resistant starch, carotenoids, and antioxidant activity, bananas collected at roughly 105 days old are the best to use in the ABB group's unripe banana flour (Moongngarm, 2014). The chemical makeup of the pulp and peel of bananas is high in polyphenol compounds and resistant starch, which protect against a number of chronic diseases, such as regional enteritis (Crohn's disease) and ulcerative colitis (UC), which are two colonic inflammatory processes that make up inflammatory bowel disease in humans (Quaglio et al., 2022). Bananas include a number of beneficial components, including the sugars glucose, sucrose, and fructose, as well as the minerals potassium, iron, and vitamin B6. As natural antioxidants, the polyphenolic chemicals in unripe banana flour would bring health advantages (Gajananrao Mahore & Shirolkar, 2018). Green banana flour that has not been peeled and has been dried on a spouted bed can be a great instrument for enhancing the nutritional value of goods and increasing their nondigestible content. Unpeeled green or unripe banana flour is notable for not producing waste, indicating the full exploitation of the fruit, enhancing output and lowering labor costs because peeling is not required, in addition to giving food items nutritional value (Bezerra, Amante, et al., 2013; Bezerra, Rodrigues, et al., 2013). The method utilized to prepare UBF (unripe banana flavor) from unripe bananas (*Musa acuminata*, var. Nanico) resulted in a highly concentrated RS and total DF. However, UBF had intact starch granules and a poor energy value. Low levels of minerals, phytosterols, accessible carbohydrates, and total polyphenols were found in UBF, which also possessed modest antioxidant activity. As a result of its carbohydrate composition, green banana product UBF shows promise as a functional ingredient (Menezes et al., 2011). Figure 1 displays a cyclical view of the main topics studied.

### 1.1 Bioactive compounds in green banana

Bioactive substances can be detected in green banana plants and extracts obtained from the pseudo stem of the banana, including catechol, pyrocatechol, tannic, gentisic, protocatechuic, ferulicas, caffeic, chlorogenic, gallic, and cinnamic acids (Saravanan & Aradhya, 2011). A solvent extraction method was utilized to obtain phenolics from antioxidant rachis (Xavier et al., 2019), which may be a valuable source of phenolics with antioxidant properties that may be used in numerous large‐scale applications (Díaz et al., 2021).

**FIGURE 2:**
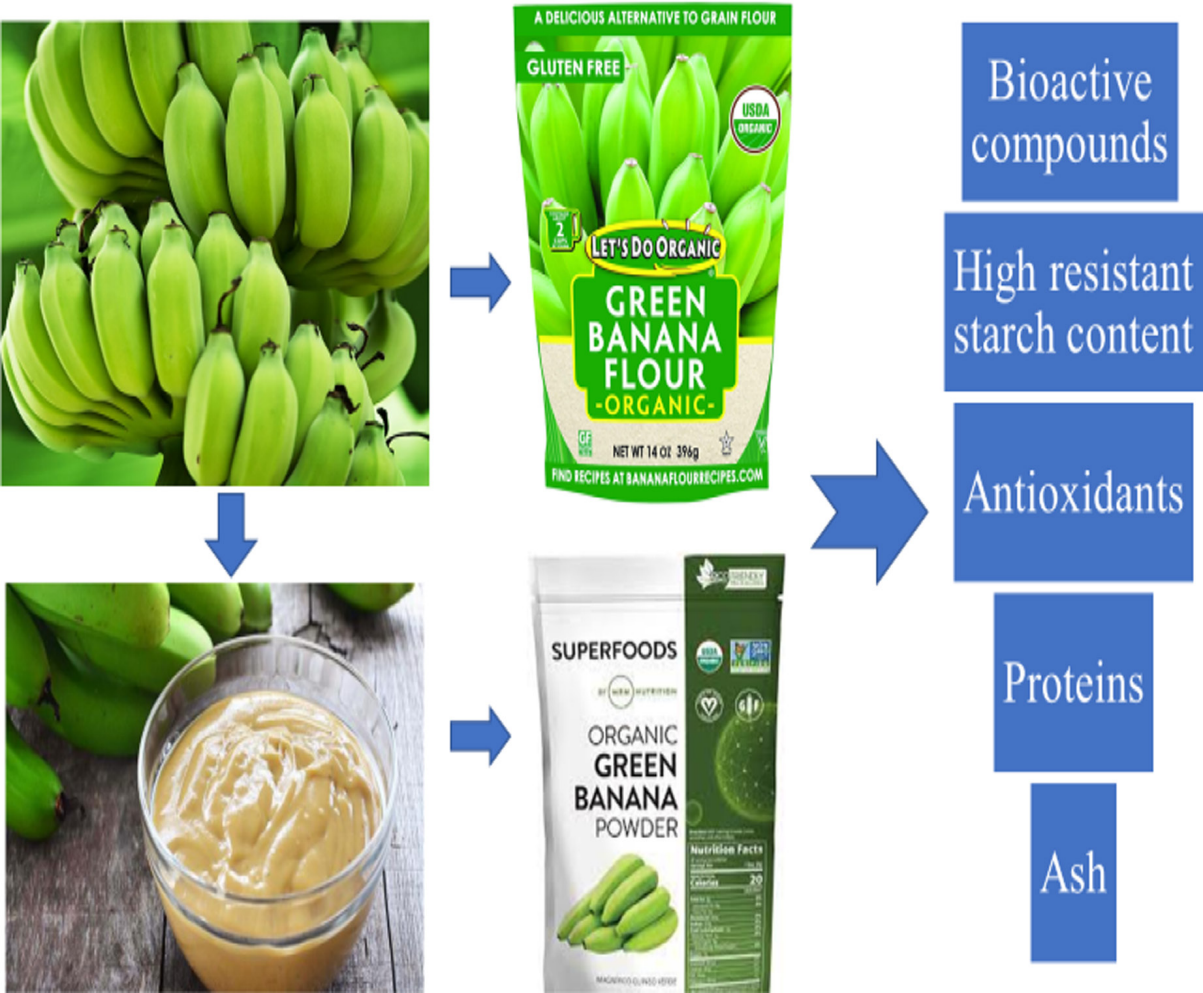
Composition of items made using green bananas.

Flavanol glycosides predominated in plantain peels, and both pulp and peel contained high levels of phenolic compounds and antioxidants. Banana rhizome, a biodegradable remnant of the banana plant, can be used as a source of antioxidants or phenolic. This has long found significant use in the food and pharmaceutical industries (Kandasamy & Aradhya, 2014). Figure 2 depicts a pictorial perspective of items made from bananas and their makeup.

### 1.2 Enzymatic role in the development of resistant starch

The studies revealed that the amount of resistant starch in green bananas flour can be appraised using an enzymatic method. Total starch content can be attributed as the sum of RDS and SDS and Resistant Starch (RS). Here, numerous modified starches or starch derivatives' structural, physicochemical, and digestibility properties were investigated. The native starch was transformed using amyl pullulanase solo pullulanase, commercial α‐amylase or β‐amylase, and malt endogenous amylase. Enzymatic processing of malt improved its amylose, RS, and thermal stability while lessening its solubility and glycemic index (Gui et al., 2022). The resistant starch in green banana flour is improved using a two‐step enzymatic process that uses amylopullulanase and amyloglucosidase to help modify the starch granules' molecular structure. The ideal values demonstrated that the greatest amount of digestible starch was converted to resistant starch (Das et al., 2022a).

To boost banana pulp's resistance to starch, undergo postharvest processing, including freeze drying. Throughout freeze drying, endogenous α‐amylases are halted, resulting in a greater concentration of resistant starch. Due to their extraordinary proportion of resistant starch, freeze‐dried banana flours either in their inherent or molten stages may aid in regulating glucose homeostasis (Pico et al., 2019).

### 1.3 Storage quality of green banana resistant starch

Evaluating the physicochemical and sensory properties of the green banana biomass to see if production and storage have any impact (GBB). By using a five‐minute pressure cooking time instead of a longer 10‐min cooking time, fiber, vitamin C, and phenolics were better protected. Between the two cooking times, there were no discernible differences in any of the acceptability traits. Although resistant starch and phenolic content were unaffected, vitamin C and fiber concentration were better retained by refrigeration than by freezing. The refrigerated GBB produced the best sensory test results in terms of flavor, texture, and all‐around acceptance. The preservation of nutrients and bioactive substances like vitamin C, resistant starch, and phenolics seems to be best achieved by storing green banana biomass in the refrigerator (Riquette et al., 2019).

Green banana flour (GBF) from Musa cavendishii, variety “Nanicao,” can be used as a functional component because of its high quantity of resistant starch (RS2). The agglomeration of GBF resulted in a larger particle size, improved immediate characteristics, and enhanced wettability, making the particles suitable for use in liquid preparations (Rayo et al., 2015).

### 1.4 Applications of green banana as a source bioactive compounds in food and pharmaceutical industry

Bananas are active in the pharmaceutical industry as a treatment for a variety of disorders because their pulp and peel both contain phytochemicals with antioxidant properties. This includes the fight against the black sigatoka disease (Tsamo et al., 2015). Studies done in the last several years suggest that eating fruits regularly will help you live longer by warding off diseases like cancer and heart disease (Wright et al., 2008). Red blood cells and preadipocytes were shielded against oxidative damage by polyphenol‐rich extracts (Septembre‐Malaterre et al., 2016).

The most common food‐sensitive enteropathy in humans, known as Celiac Disease, is caused by consuming wheat gluten and related proteins in barley, rye, and oats. Green (unripe) bananas are incredibly nutrient‐dense and advantageous to one's health, including the polysaccharides' antioxidants and polyphenols, as well as potassium, essential minerals, and vitamins like provitamin A, carotene, B1, B2, and C (Rebello et al., 2014). These characteristics allow it to be used in the manufacture of gluten‐free cakes, muffins, and cookies (Türker et al., 2016).

The conjugates of biomacromolecules like polysaccharides and proteins may be able to stabilize the emulsion system. Native banana starch was extracted from green culled bananas using this method, and the resulting lintnerized‐autoclaved banana starch was produced (LABS). Soy protein isolate (SPI) was used to create protein‐polysaccharide conjugates. It has been demonstrated that the LABS‐SPI conjugate emulsion system increases the stability of the bioactive component astaxanthin, which is used to biofortify foods and medicinal formulations (Shrestha et al., 2018).

### 1.5 Green banana resistant starch commercial products

Some examples of green banana flour sold commercially are NuBanaRS65, green banana resistant starch, and organic green banana flour (Walsh et al., 2022). Supplement powders, bars, beverage powders, cold‐fill drinks, and raw vegan meals may all benefit from using NuBanaRS65, which contains Minimum 65% RS2 Prebiotic fiber, is non‐GMO, and is not hygroscopic (International Agricultural Group, 2018).

The second commercial product, green banana resistant starch, is in fact a blend of soluble, insoluble, and fermentable fiber. It has no preservatives or additives, is vegan, paleo, gluten‐free, and high in fiber. It is free of gluten, vegan, paleo, rich in fiber, and has no additives or preservatives. Smoothies, cereal, soup, water, juice, and other foods may all benefit from the addition of this ingredient because it is rich in inulin, zinc, magnesium, phosphorous, and manganese (Natural Evolution, 2019). Finally, organic green banana flour made from dried and milled organic green bananas. It is organic, does not contain any GMOs, and is a Source of RS2. It is devoid of gluten and grains. suitable for paleo and keto diets that use natural sweetener. Prebiotic DF and RS source, essential amino acid source, high‐fiber replacement for all‐purpose white flour. Using them in baking and cooking Maybe added to smoothies (Live Kuna, 2021).

### 1.6 Green banana resistant starch as fat replacers

The studies showed that the green banana biomass (GBB) contained 4.16% resistant starch in addition to the good amount of dietary fiber and antioxidant potential. When used as a fat addition in chicken mortadella, green banana biomass (GBB) has the potential to improve sensory perception (Auriema et al., 2021). Due to customer demand for healthier diets, a reduction in the fat level of processed beef products has become significant. In this respect, the resolution of this research was to provide a description of chicken mortadella. with entire and partial replacement of chicken skin with green banana biomass (GBB). Mortadella's WHC increased when GBB was substituted with 100% chicken skin, all while maintaining the ES. It is consistent with the flavor of mortadella to say that the flavor of the samples was dominated by the fibrous and fat components without any noticeable change from the addition of GBB. Based on the findings, it is conceivable to replace up to 100 percent of chicken skin with GBB to produce a healthier blended meat product with good sensory acceptability (Auriema et al., 2022).

Poultry meat is one of the most consumed animal meals on a worldwide scale, and its consumption and processing have expanded dramatically over the past several decades. Cooking unripe bananas (stages 1 and 2 on the Von Loesecke (1950) maturity scale) yields green banana biomass (GBB), a byproduct of banana handling. The pulp from a green banana has nutritional value in addition to not having any flavor or aroma when cooked, making it a useful technical ingredient in a range of cuisine recipes (Izidoro et al., 2008; Ranieri & Delani, 2014). Despite this, there is little research on GBB's ability to replace fat in beef by‐products (Alves et al., 2016; Bastos et al., 2014; Dinon et al., 2014). The GBB's physicochemical properties, antioxidant potential, and antibacterial activity were assessed.

The formulations' microorganism‐resistance was judged, and the sensory profile was assessed using the Preferred Attribute Elicitation (PAE) method. The results suggested that GBB might be used as an efficient component, a source of resistant starch, a mineral contributor, and a source of vitamin C. Moreover, green banana biomass extract exhibited antimicrobial and antioxidant properties and may have helped emulsion stability and water croft capacity, which are desired characteristics in emulsified meat products. These results led to the conclusion that GBB demonstrated advantageous operational and technical protocols to be used in chicken mortadella without altering the traditional flavor of these products (Bruna Emygdio Auriema et al., 2021).

The functioning of the variously constructed requeijao cremoso processed cheeses was maintained by the inclusion of encapsulated probiotic microbes over the forty‐five days of storage. GBB reduced fat and protein but increased moisture and water activity when used as a partial fat replacement. Both the fat and the GBB contents had an effect on the texture. Findings suggest that GBB and encapsulated probiotic bacteria might be employed in the preparation of requeijao cremoso treated cheese for dairies, hence boosting the functional merit of this product (Pivetta et al., 2019).

### 1.7 Effective utilization of green banana starch

Because of their low retrogradation rate, low swelling characteristics, and strong resistance to heat and amylase occurrence, banana starches are utilized in the food industry as a gelling, thickening, and stabilizing ingredient. Green banana pulp (GBP), which is significant to the dairy industry, can be used to make a variety of desserts because it is odorless and soluble (Padam et al., 2014). Unripe bananas (*Musa acuminata*, var. Nanico) have a high concentration of RS and total DF when they are peeled, which results in UBF. UBF also included few calories and undamaged starch granules. Low levels of minerals, phytosterols, accessible carbohydrates, and total polyphenols were found in UBF, which also had modest antioxidant activity. Overall, UBF appears to have the potential to serve as a functional element due to its carbohydrate profile (Menezes et al., 2011).

The starch found in banana waste is a valuable source for the textile, paper, pharmaceutical, food, and polymer industries, among others. Large amounts of waste are produced during banana production that could be converted into natural bioactive compounds and more valuable products (Ahmed, 2020). Functional foods are increasingly being included into the confectionery industry in an effort to satisfy consumer demand for products that are both aesthetically pleasing and nutritious. The icings made from flour and green banana biomass had lower overall calorie counts and higher concentrations of resistant starch, and protein, making them healthier. In addition to being more intensely colored and softer than commercial varieties, these icings also had good sensory acceptance, particularly the chocolate flavor, which received more than 72% of the score. For the purpose of making functional foods, bananas with a low amylose level are useful (Lehmann et al., 2002). Banana flour acquired using Refractance Window drying was found in another investigation to preserve more of its qualitative characteristics than banana flour obtained through hot‐air oven drying. Due to its nutritional properties, UGBF may be generated by RW drying for use in different gluten‐free baby meals, confectionery and bakery goods (Padhi & Dwivedi, 2022).

Extruded UBF may be used in instant beverages and foods marketed to youngsters and the elderly since it was more water soluble (Naivikul & Arlai, 2022). At various screw speeds, mango and banana extrudates showed less distension than corn starch extrudates. Based on the starch supply and the screw rate, the extrudates' solubility varied. Mango and banana extrudates exhibited similar resistant starch levels when made at 30 rpm, whereas banana extrudates had greater RS when prepared at 40 and 65 rpm. The levels reached in the extruded products make the use of unusual starch sources a viable option for resistant starch manufacturing. The extrusion circumstances have a noteworthy impression on the functional and physicochemical properties of RS products, making them a crucial aspect in their production. Utilizing starch extrudates in medicinal and food applications may be a significant area of growth (Gonzalez‐Soto et al., 2006). According to the research RSRP from banana starch as a possible additive for bread items comprising poorly digesting carbs (Aparicio‐Saguilán et al., 2007). Ultrasound therapy is a recently established technology based on the propagation of ultrasonic waves and is regarded as a novel method. This study expanded the commercial use of green banana starch by improving its solubility, swelling power, water binding or immersion capacity, rheological qualities, and gelatinization (Izidoro et al., 2011).

Green banana flour normally has a high viscosity and a strong tendency to rearward; these two characteristics are important for its use as a thickening agent. The maintenance of this article limits the use of starch products with rapid qualities, but expands their use as the foundation for sauces, puddings, and flans when a higher viscosity is preferred (Bezerra, Amante, et al., 2013; Bezerra, Rodrigues, et al., 2013). Staphylococcus coagulase‐positive development was not observed in samples of green banana flour that had undergone 1 and 3 kGy of radiation exposure.

**FIGURE 3:**
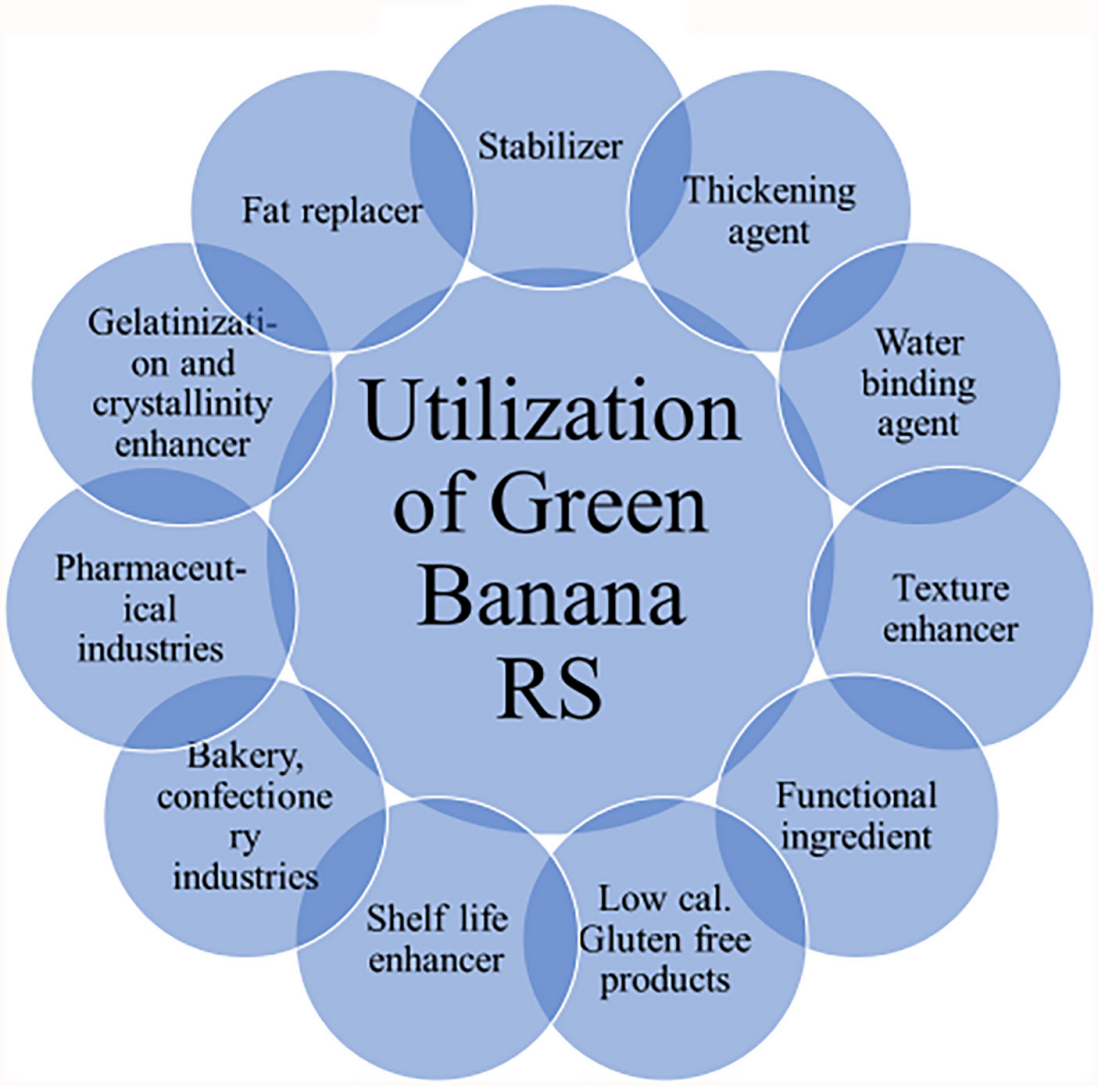
Effective utilization of GBRS.

Additionally, it was found that both dosages of irradiation were efficient at reducing the microbial load of mesophiles. Therefore, the use of irradiation to improve food safety may be advocated (Taipina et al., 2013). Substituting unripe banana flour for rice flour considerably boosted the RS content of rice noodles without affecting their acknowledgment among panelists. Consequently, the synthesis of green banana flour and its use in the enlargement of functional foods namely rice noodles in this study that are rich in resistant starch is prospective, and this product is likely highly nutritious for individual fitness (Moongngarm, 2014). Utilization of GBRS indicators shown in Figure 3.

### 1.8 Application of green banana in baking industry and confectionery

Green banana flour, added at 15% and 20%, makes for a sliced bread that is rich in taste and texture and has a resistant starch content that is more than four times as high as that of conventional sliced bread. Gluten‐free and abundant in indigestible components such resistant starch and nonstarch polysaccharides, green bananas are a starchy food with a great concentration of these substances (Kaur et al., 2017). By combining flours of rice and green banana peel in agreement to the quality criteria suggested by the Brazilian legislation (Sotiles et al., 2015), gluten‐free products like cakes and muffins free of gluten may be made (Kaur et al., 2017; Türker et al., 2016). Banana flour's prolonged shelf life was explained by its low moisture content, according to proximate analysis (Malindi, 2021).

Banana is a great origin of nutritional fiber that may be utilized in food preparation. Using banana fiber only or in conjunction with ice cream stabilizers verified effective in the creation of ice cream. Nutritional and physiological qualities can be improved by fortifying ice cream with green banana pulp and peel flour (Yangılar, 2015).

### 1.9 Other food products

The glycemic index of Asian noodles is high, and an imbalanced diet strong in carbs has been related to obesity and overweight. The purpose of green banana flour is to produce low‐calorie gluten‐free noodles (Yu et al., 2020) and pasta with enhanced nutritional value (do Nascimento Tinoco et al., 2022). Banana starch has demonstrated potentiality for use in an array of culinary applications (frozen and refrigerated meals, soups, and pates, and the production of sauces sector), suggesting its potential as an alternative technology and creation of food items (Fontes et al., 2017). As a result of adding 1–3 percent green banana flour to milk, the fermentation process yields a product with enhanced flavor and aroma (Batista et al., 2017).

### 1.10 Green banana RS usage in biofilms and edible packaging

As a biodegradable polymer, starch is frequently combined with other polymers to create films and lessen degradation issues associated with synthetic goods. Films used in the food sector need to meet specific criteria, such as being transparent and resistant to heat (Rodríguez‐Ambriz et al., 2008). The employment of banana starch in the formation of biofilms and as a mechanism of nutritional enrichment and viscosity enhancement in foods subjected to heat processing. According to research employing banana starch, starch modifications can be made to enhance the physical and hurdle properties of the films that are produced (Zamudio‐Flores et al., 2006). The viscoelastic properties of the filmogenic suspension were enhanced by a higher amylopectin content and decreased by a lower concentration. Green banana flours are a potential option for its industrial applications due to their ability to produce filmogenic solutions (Salazar et al., 2022).

Banana starch film's high level of transparency makes it aesthetically pleasing and improves customer acceptability of certain items. Starch film with a high glycerol concentration might be used to package light‐sensitive foods like fruits and rolls (Alimi et al., 2022). One of the reasons of postharvest decay in fruits is water loss. Due to their improved handling and reduced stiffness, films produced from banana starch and copolymers were chosen to investigate their possible application as a strawberry covering.

### 1.11 Adulteration factor in green banana products

Wheat adulteration is a serious nutritional concern for consumers and a potential danger to the manufacturing along with its distribution of green banana flour. Vis–NIR showed to be a valuable technology that elucidated the structural characteristics of the differences in banana flour starch and further contributed to the reduction in resistant starch (RS). It was demonstrated to be a cutting‐edge quality indicator for determining the reliability of GBFs. It is anticipated that the variations brought on by various additions of wheat may be used to quantify the level of adulteration, give manufacturers direction as they create new green banana‐wheat flour composite goods, and ultimately lessen the harm wheat adulteration has caused to the food industry (Ndlovu et al., 2021).

## 2 FUNCTIONAL PROPERTIES OF GREEN BANANA RESISTANT STARCH

### 2.1 Thermal property

A high‐pressure Differential Scanning Calorimeter (DSC) was used to define the thermal properties (Kongolo et al., 2017). After subjecting green banana starch to a heat and moisture treatment in the existence of citric acid (CAHMT), its morphology, pasting, and thermal characteristics were examined under a wide range of temperature. The CAHMT banana starch injection may reduce body fat accumulation and alter the gut's microbiota. Using the CAHMT technique at 90°C, banana RS's thermal stability was enhanced, creating new opportunities for its application in nutritious meals. CAHMT successfully elevated green banana RS's thermal stability. The gelatinization and solubility temperatures were also increased, and the relative crystallinity of the treated banana starch was also improved. The crystalline X‐ray diffraction pattern shifted from B‐type to A‐type. Green banana starch that has been processed may be employed in a number of culinary applications that call for a high dietary fiber content and heat processing due to its increased thermal stability and physiological activities (Wu et al., 2020). The most nutrient‐dense banana flour with the least amount of RS content lost was produced when banana slices were dried at 55oC, according to an analysis of the effect of drying temperature on the drying model quantities (Kumar et al., 2019).

### 2.2 Pasting property with and without banana peel

The sample's pasting qualities were evaluated using a Quick Visco Analyzer (Udomkun et al., 2021). As comparison to rice flour and wheat flour, banana flour had greater peak, hot paste, cold paste, and setback viscosities. The reverse viscosity, which has been connected to product texture, is a reflection of the amylose reassociation or retro degradation of starch after cooking (Detchewa et al., 2021). It is known that banana flour has unique pasting and textural properties, as well as significant levels of resistant starch and dietary fiber. After fat, amylose, and carbs as test variables, protein content had the biggest effect on the quality of the complete paste. Increases in protein, fat, and amylose content significantly lowered peak and ultimate viscosities, whereas increases in carbohydrate content had the reverse effect. Yet when peel was added to banana flour, the end result was better pasting properties. The blended peel sample had slightly lower pasting temperatures, peak viscosities, breakdowns, ultimate viscosities, and setbacks (Kongolo et al., 2017).

### 2.3 Solubility

After fat, amylose, and carbs as test variables, protein content had the biggest effect on the quality of the complete paste. Increases in protein, fat, and amylose content significantly lowered peak and ultimate viscosities, whereas increases in carbohydrate content had the reverse effect. Yet when peel was added to banana flour, the end result was better pasting properties. The blended peel sample had slightly lower pasting temperatures, peak viscosities, breakdowns, ultimate viscosities, and setbacks (Kongolo et al., 2017). Green banana flour's poor solubility prevents it from being widely used in instant foods; to improve its solubility, it would need to undergo several treatments, such as gelatinization and subsequent starch solubilization. Due to structural and morphological modifications, extruded powders of CBF (commercial banana flour) are more readily soluble in water. The maximum solubility values (about 80%) were discovered for beginning barrel temperatures between 40 and 50°C with a screw speed of 800 rpm (Giraldo‐Gómez et al., 2019). The particle size distribution of the flour used has a considerable impact on the functional qualities and end product quality. Results from measuring WSI across a wide variety of particle sizes indicated that UBF was not hydrophilic. Very high wettability in other particle ranges demonstrated superior reconstitution characteristics. WSI values across the board for different sized particles were rather low, indicating that UBF was not soluble in water. The maximum solubility values (about 80%) were discovered for beginning barrel temperatures between 40 and 50°C with a screw speed of 800 rpm (Giraldo‐Gómez et al., 2019). The particle size sharing of the flour used has a considerable effect on the functional qualities and end product quality.

### 2.4 Water binding capacity

Dough microstructure was disrupted to variable degrees by the RS2‐Zn system, which reduced water absorption, moisture content, and molecular mobility. The degree to which water is bound in dough depends on the shape of the protein, the flexibility of the binding of RS2‐Zn, and the water. As a result, substrates with variable degrees of stiffness, flexibility, and water‐holding capacity are produced as a result of RS2‐Zn‐ability carbonates' ability to change the protein's final spatial arrangement (Qin et al., 2021). The particle size of the unripe banana flour, or UBF, had a substantial impact on how well the MP composite gel worked. However, by improving water retention and texture, UBF might be a useful component that improves the rheology and microstructure of the final MP composite gel system (Pereira et al., 2020). Green banana flour was formed using five distinct varieties of green banana, including Grand Naine (AAA), Monthan (ABB), Saba (ABB), Nendran (AAB), and Popoulu (AAB) (GBF). Monthan flour performed better than Popoulu flour at 70–90°C in terms of swelling and water‐holding capacity, whereas Popoulu flour performed better in terms of solubility. WHC g (g/100) is the result of dividing the sum of the dry residue's weight by the weight of the wet residue. The water‐holding capacity of green banana flour is resolute not only by the dietary fiber, resistant starch, protein, and amylose but also by the physical properties of the starch granules. This information in the creation of a more expansible matrix, which in turn results in an increased water‐holding capacity. Banana starch granules have the right form and size to be used as a raw material for making edible films with a larger water absorption capacity and quicker disintegration, in addition to its many food‐related uses. The water retention capabilities of the samples of banana flour that were dried using the freeze drying method were considerably greater. Freeze‐dried banana flour was found to have a larger percentage of resistant starch (46.72 percent) (Khoozani et al., 2019). Wheat flour's functional qualities, such as bulk density, oil holding capacity, and water‐holding capacity, were increased when muomva red banana flour (MRF) was added to the mix. As MRF content rose, it was discovered that all flour gelatinization temperatures increased (Mabogo et al., 2021). Due of its morphological features and apparent relationship with high WAC, GBF starches may find value in the development of edible films. All of the GBF were establish to have relatively high RS, making them excellent runners for use in the formulation of low‐GI food items. Different types of green banana flour, including Grand Naine, Psiang A Wark, Finger Rose, FHIA‐01, and Du Roi, have variable amounts of resistant starch and water absorption ability. The Finger Rose cultivar showed the lowest WAC, although the Pisang Awak variety recorded the highest. The high WAC reported with Pisang Awak GBF in this study indicates that it may be acceptable for baking. A lower WAC is preferred for a diluter consistency, since it effects gelatinization via accessible water. The FHIA‐01 had the most resistant starch, whereas the Grand Naine had the least (Khoza et al., 2021) (Table 1).

**TABLE 1:** The functional properties of typical green banana flour under certain processing conditions.

Parameters	(%) Range
Moisture content (Xw)	3.75–6.51
Water activity (aw)	0.333–0.483
Wettability (tW)	8.5–56.5
Solubility (S)	59.36–80.61

### 2.5 Crystallinity

Considering that a polymer's crystallinity can be used to estimate how many of its areas are crystalline and how many are amorphous. An exothermic reaction that results from the development of crystallization in response to heating can be used to measure the degree of crystallinity (Paul et al., 2015). The ODF50 (oven‐dried samples) of banana flour had the highest levels of amylose content and crystallinity. 50°C is the best drying temperature for hot‐air ovens, with no impact on RS content, according to analysis of the high crystallinity and gelatinization temperatures of starch (Khoozani et al., 2019). B‐type crystals and broken starch granules were found in banana flour (Chang et al., 2022).

### 2.6 Gelatinization

**FIGURE 4:**
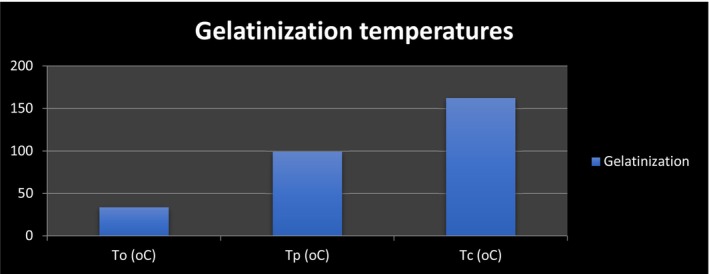
Average values for the optimum temperature (*T*_*o*_), peak temperature (*T*_*p*_) and temperature of completion (*T*_*c*_) in degree Celsius for GBRS.

Bananas had a significant amount of carbohydrate having 91.11–96.24 percent; (dry basis), but only trace amounts of protein (0.16–0.31), fat (not identified), and ash with 0.25%–0.35%. Gelatinization enthalpy (H) extended from 9.8 to 12.3 J/g, while amylose concentrations were between 25.89 and 33.48 percent and the transition temperature was between 63.89 and 86.83°C (Samanros & Lin, 2021). The gelatinization temperature of green banana starch was found to be rather high, at 73.37°C (Coutinho et al., 2021). Higher levels of amylose and crystallinity were associated with a higher gelatinization temperature. Its elevated gelatinization temperature has been attributed to a rise in crystallinity (Das et al., 2022b). Native starch granules from certain plant sources, such as green bananas and potatoes, make up Type II RS. Because of their compact structure, undamaged granules are incapable of being gelatinized; this prevents enzymatic digestion from taking place. Amylose and starch that have been retrograded are what make up type III RS. Due to its linear shape, amylose is more likely to form double helices when exposed to conditions that produce this phase transition, such as a temperature of around 4°C and an acceptable amount of moisture. In addition, cooking is ineffective in dissociating the retrograded amylose, which has a gelatinization temperature of up to 170°C (Thuy et al., 2022). RS has great potential in the food processing zone as an all‐natural dietary fiber due to its excellent safety, high gelatinization temperature, and low viscosity (Wang et al., 2020) (Figure 4).

### 2.7 Foaming

The amount of foaming capacity was determined by calculating the percentage increase in volume. Foaming capacity is essential for defining the skill of flour to foam, which is dependent on the presence of flexible protein molecules that lower the surface tension of fluid. In comparison to other flours, unripe green flour has a greater capability for foaming. The talent of flour to foam is a crucial factor in determining whether it may be used as a whipping constituent in whipped foods (Mashau et al., 2022). Unripe banana powder was created by the foaming‐mat drying procedure. The chunkiness of the foam had an impact on the physical, structural, and warm air properties of the powder. Unripe banana powder (UB) dried on a foam mat can be used as a foundation of fiber with prebiotic potential in the baking and confectionary areas to make useful products like functional cake as of its high‐resistant starch content (Kamali et al., 2021).

### 2.8 Bulk density

A sample of flour's bulk density can be thought of as a measurement of its heaviness. It is a typical that establishes the weight of packaged goods, principally flour produce. This is helpful for loading and carrying packages from one place to another. It suggests a larger quantity of unripe banana flour resistant starch be packaged. Pretreatment of unripe green banana flour with ascorbic acid produced the maximum bulk density, whereas pretreatment with lactic acid made the minimum (Anyasi et al., 2017).

### 2.9 Effect of GBRS on the texture of flour confectionery

#### 2.9.1 Biscuits

The majority of taste testers preferred biscuits made with a flour blend containing 50% unripe Saba banana flour and 50% wheat flour. Therefore, the use of USBF in biscuits substantially increased the dietary fiber RS, and ash content, while drastically lowering the protein, moisture, and hardness, while still remaining within the threshold of a good quality biscuit (Ayu et al., 2021). When kept properly, biscuits may last much longer than other baked items (Senanayake et al., 2021). Through a compression test, we had able to ascertain the biscuits' hardness and factorability. Gluten‐free biscuit doughs improved with RS have been observed to have a low hardness. RS has the capacity to bind water, leading in a dough that is more elastic. The lowest hardness was seen at the highest RS level. The inclusion of RS had a softening impact on the texture of dough and biscuits and led to the decline of dough stickiness (Di Cairano et al., 2021).

#### 2.9.2 Bread

The bread was denser, firmer, and chewier when made with whole green banana flour. Large deformation characteristics of dough were found to be connected to its textural qualities. 30% substitution of wheat flour with green banana flour formed a fortified loaf of bread. Compared to wheat bread, the crumbs from the fortified bread samples were much tougher and chewier (Khoozani et al., 2020).

#### 2.9.3 Crackers

The crispiness of crackers relies heavily on their low moisture content due to the rapid removal of water during the frying process. Oiliness and a mouthfeel texture are not the only drawbacks to frying, though; high calorie counts are a byproduct of the oil being absorbed to replace part of the water that was lost. There are two physical characteristics that are associated with the crispiness of the crackers: the bulk density and the expansion ratio. Crispiness resulted from a combination of a high expansion ratio and a low bulk density, both of which indicated a high level of structural porosity (Say et al., 2021).

#### 2.9.4 Gluten‐free rice cookies

The grain rice (*Oryza sativa* L.) does not contain gluten. The resistant starch concentration of unripe banana flour was pretty high. It validated the viability of substituting unripe banana flour for rice flour in the production of gluten‐free rice‐based cookies with enhanced texture, color, and high acceptance, on par with wheat flour cookies. Gluten‐free rice‐based cookies can have up to 70% of the rice flour substituted with unripe banana flour. As a result, rice cookies made with high‐resistant starch and no gluten might be developed as an useful food item (Detchewa et al., 2021).

#### 2.9.5 Muffins (gluten free)

Due to their cheap production cost related to other gluten‐free goods and excellent sensory appeal, high protein digestibility, and excessive antioxidant potential, muffins prepared with green banana flour are a feasible option to gluten‐free healthy food (Radünz et al., 2021). The muffins' firmness and springiness were tested using the texture analyzer. Adding green banana flour made the muffins more substantial. A bigger rise in value is associated with a higher fiber content. The crusts and crumbs in the muffins were darker due to the fibers' added natural color. Due to its improved water binding capacity, GBF significantly decreased baking losses in the muffins (Harastani et al., 2021).

#### 2.9.6 Cakes

Cakes with layers and sponges that were developed using green banana flour instead of corn flour. The goal was to establish the appropriate substitution proportions in order to generate nutritionally enhanced goods while maintaining texture and sensory features that were desirable. Sponges and layer cakes lost some of their cohesion and gained some of their hardness when green banana flour was used in place of 30% of the corn flour. The sensory qualities and texture aspects of sponge cakes were enhanced by the addition of up to 30 percent GB flour (Segundo et al., 2020).

#### 2.9.7 Noodles and pasta

The noodles made with unripe banana flour managed better than those made using wheat flour (Balmurugan et al., 2022). Improved nutritional value and sensory qualities are achieved by combining BF and hydrocolloids in noodle products (Tangthanantorn et al., 2021). Dough with the right amount of strength and extensible texture may be made by combining wheat flour and resistant starch source flour (10–20 percent, w/w), like the GBF, so that salty noodles can maintain their stiff and elastic form. The incorporation of resistant starch cause flour with salted noodles increased the nutritious content and decreased the glycemic index (Li et al., 2022).

#### 2.9.8 Macroni

A prebiotic dietary fiber called type two resistant starch gives macaroni special technical advantages in its production. At a 10% concentration, RS2 dramatically reduced the adhesiveness and chewiness of cooked macaroni while accumulative its hardness and cohesion. It is possible that resistant starch type 2 might serve as a dietary fiber, with the added benefit of being able to improve the product's texture, all without having an adverse effect on customer acceptability. Benefits to metabolic profiles might be gained from eating banana RS4 macaroni (Chuathong et al., 2021).

### 2.10 Effect of GBRS on the texture of meat (sausages)

Banana flours (pulp, peel, or whole banana) work well in place of wheat flour when preparing Frankfurt‐style sausages. Because banana starch has a pH‐neutralizing effect, the pH of the sausage does not change during storage, and the pH shift has no impact on the texture of the sausage. Banana peel flour's high fiber content encourages water retention, increasing the product's hardness. Similar to this, the resistant starch present in banana flour could aid in the sausage's ability to hold onto moisture and impart a superior texture (Salazar et al., 2021).

### 2.11 Effect of GBRS on the texture of dairy products

When green banana starches were introduced at increasing quantities, the firmness of fermented goods derived from baru almonds increased, syneresis reduced, and the pH, titratable acid, soluble solids, and color all improved. Starches from green bananas can be used to achieve natural thickening. The fermented baru product made from almonds that contains 4.5% green banana starches stands out since it promises to be both a probiotic snack and a dairy substitute (Coutinho et al., 2021).

### 2.12 Shelf life of GBRS

Bananas are an agricultural product that is perishable. Most bananas are eaten raw, whereas only a small percentage are used in processed foods. Bananas have a low shelf life because of the intrinsic trait that they possess. Another option to extend bananas' shelf life is to turn them into other food items through processing. Bananas may be ground up into flour as one example of these measures. Green banana flour's moisture content can be reduced by processing (Wibowo et al., 2021). Consequently, the GBF's comparatively low moisture content may make it stable and provide it a longer shelf life (Khoza et al., 2021).

### 2.13 Calorific value and cholesterol content of GBRS

Higher quantities of ash, fiber, resistant starch, and protein were found in green banana biomass and flour, whereas total caloric value was reduced in icings (Silva et al., 2021). The percentages of ash, crude fiber, and total carbohydrate in cookies all increased as the UBF went up, whereas the percentages of crude fat, crude protein, and calorie content went down (Taleb & Arafa, 2021). Gluten‐free biscuits with a lower glycemic index and lower calorie content may be possible by completely substituting sucrose with resistant starch or banana four, in addition to pseudo cereal and legume four. However, resistant starch in a formulated form may have a number of positive health effects, such as controlling fasting plasma triglyceride and cholesterol levels and enhancing glycemic regulation (Di Cairano et al., 2021).

## 3 HEALTH PROPERTIES OF GREEN BANANA RESISTANT STARCH

### 3.1 Immunity

Banafine a registered vaccine was developed as a food powder by the enzymatic hydrolysis and fermentation of green, unripe banana powder. Green bananas are unfit for human eating due to their astringent and bitter flavor (Liao & Hung, 2015; Zandonadi et al., 2012). The unripe Cavendish bananas were treated with amylase and protease, and then yeast fermentation was used to generate the novel banana powder banafine. Since the harsh and harsh flavor of unripe green banana was removed, banafine has a far upgraded taste. The methods described here not only improved the flavor of unripe green bananas, but also encouraged the immune system by causing the release of several cytokines. Furthermore, empirical evidence obtained from mice show that ingestion of banafine displays immunomodulatory action, which would be advantageous for the prevention of influenza infections and the management of those that already exist. This study exposed the potential application of unripe green banana as a functional food ingredient, which is advantageous for sustainable agriculture (Horie et al., 2020).

### 3.2 Diabetes

Diabetes mellitus is a compound set of metabolic abnormalities linked with microvascular problems due to hyperglycemia and other metabolic diseases, indicating an increased risk of emerging cardiovascular disease (CVD). Controlling risk factors, such as strict glycemic management, lowers macro‐ and microvascular problems (American Diabetes Association, 2017; Garber et al., 2017; Ogurtsova et al., 2017). Green banana flour is a possible functional food for managing diabetes since it has the ability to improve glycemic control and intestinal health (da Silva et al., 2014; Rastall & Gibson, 2015). Green bananas' high RS content has been linked to a number of health benefits, such as easing constipation, controlling diabetes and obesity, and preventing colon cancer. Molecular structure had an impact on in vitro digestibility, and results revealed that banana flour, which included a lot of the starch amylose, was difficult to break down (Bi et al., 2017). The in vitro digestibility of banana flour starch was measured across four maturity levels. Banana flour from the first ripening stage had the most noticeable impact. Banana flour may influence mRNA expression levels of genes involved in glucose metabolism, leading to increased hepatic glycogen production and decreased gluconeogenesis and glucose output. Using natural banana flour could be helpful in the treatment of type 2 diabetes (Yang et al., 2020). Different sources of RS have been shown in short‐term clinical trials to enhance glucose metabolism, anthropometric parameters, and postpone the onset of diabetes and its consequences. Our findings, together with the length and scale of our trial compared to others, suggest that RS derived from green banana biomass may be a cost‐effective and appealing choice for people at risk for developing diabetes. The writers (Costa et al., 2019). Cookies made with UBF had a different chemical makeup than those made without, with a lower protein concentration and a greater dietary fiber content. The resistant starch content of cookies made with UBF was shown to rise significantly. The estimated glycemic index and reduced hydrolysis percentages also reflect this. This provides a nutritionally viable option for those with diabetes (Agama‐Acevedo et al., 2012). Intervention with UBF (about 15 g of Resistant starch per week) had a beneficial effect on glucose homeostasis, as showed by a reduction in serum fasting insulin secretion and improvements in the insulin resistance markers HOMA2‐IR and QUICKI. UBF, which contains a lot of RS, can be utilized as a functional ingredient to make more goods with a high RS content more widely available, which may help people adopt healthier diets overall (Hoffmann Sardá et al., 2016). Green banana and edible canna flours were found to have the highest concentrations of RS among the flours studied. These compounds also shown promise as functional additives for reducing GIs in starchy food items (Srikaeo et al., 2011).

### 3.3 Gut wellness

The chemical makeup of bananas, which is based on glycosylated polyphenols and resistant starch, is linked to their beneficial effects on the stomach. Improved epithelial barrier function, changed intestinal microbiota, improved production of short‐chain fatty acids (SCFAs), and improved immunological response are just a few of the processes through which these advantages manifest (Quaglio et al., 2022). Banafine containing AGC may be able to maintain intestinal health (Horie et al., 2020). According to Boulangé et al. (2016), dysbiosis and the detrimental effects it has on the GI tract, including bacterial proliferation and translocation and the breakdown of the intestinal barrier, are frequently linked to obesity. Green banana eating has been related with improvements in gastrointestinal symptoms and diseases, glycemic and insulin metabolism, body weight controlling, and the hepatic and renal effects of diabetes (Falcomer et al., 2019; Lousek et al., 2021; Rosado et al., 2020). Significant improvements were seen in the inflammatory state, adipose tissue remodeling, and the composition of the gut microbiota in male rats fed a diet containing 15% GBF. GBF consumption improved the metabolic health, adipose tissue remodeling, and gut microbiota composition of obese mice while reducing edema (Rosado et al., 2021). Assessing the impact of unripe banana flour (UBF—48% resistant starch, a prebiotic) on serum concentrations of IS, pCS, and IAA in peritoneal dialysis patients (PD). Indoxyl sulfate (IS), p‐cresyl sulfate (pCS), and indole 3‐acetic acid (IAA) are some metabolites generated from the gut, and their buildup has been linked to the severity of chronic kidney disease (CKD). Only those people who were able to consume 21 g/day of the UBF had a reduction in IS (de Andrade et al., 2021). Combination treatment of B. coagulans and GBRS synbiotic, which have been shown to have useful effects on gastrointestinal health, acted synergistically to reduce swelling in model of inflammatory bowel disease. The research gives new perceptivity into the synergistic processes of synbiotic supplementation that role by resolving dysregulated immune reaction, reduced mucosal barrier integrity, and changed metabolic profile, hence decreasing gut swelling. The fact that the synergistic effect lessened or stopped the severity of the disease in the DSS‐induced rats model supports more research into how to reduce inflammation in human IBD (Shinde et al., 2020). Constipation in children and adolescents can be effectively treated with laxatives, but adding green banana biomass to this treatment can dramatically accelerate symptom alleviation, according to an evaluation of the efficacy of combination treatments (Cassettari et al., 2019). Concentrations of acetate, propionate, and butyrate were all raised after a diet that included green dwarf banana flour either at five or ten percent. Reduction in lesion extension, suppression of myeloperoxidase activity, prevention of glutathione depletion, improved mucin synthesis, and mucosal healing were further indicators of the protective effects. In this manner, dietary green dwarf banana controls oxidative stress and colonic synthesis of SCFAs, so improving intestinal tissue protection, and may be utilized as a supplement to stop or avoid return of symptoms in inflammatory bowel illnesses (Almeida‐Junior et al., 2017). The findings from the bioaccessibility analysis showed that Mg and Mn were the most bioaccessible elements in the GI tract, followed by Zn and Cu, with Ca and Fe having the lowest bioaccessible proportions. Further, several critical elements exhibited moderate bioaccessible proportions, suggesting that green banana flour might be used to supplement the human diet; for example, magnesium (Mg) displayed RDI contributions of 15% and manganese (Mn) above 50% (do Prado Ferreira & Teixeira Tarley, 2020). By functioning as a prebiotic, green dwarf banana flour decreases intestinal inflammation, and the synergistic benefits of prednisolone are further enhanced when taken with this dietary supplement. To do this, we used the rat colitis trinitrobenzenesulfonic acid (TNBS) model. A diet containing 10 percent of green dwarf banana flour in conjunction with prednisolone provided additional dynamic protective effect than either prednisolone or a banana flour‐based diet of 10%–20% alone. Dietary supplements and complementary medicine products containing green dwarf banana flour have been shown to be efficacious and protection of inflammatory bowel disease in humans (Scarminio et al., 2012). The ileostomy model is now the gold standard for determining RS in vivo. The accretion of RBF with dominant concentration of RS2 had no effect on the excretion of further nutrients or total sterols, with the exception of a modest boost in iron excretion, and may have distinct impacts on colonic fermentation (Langkilde et al., 2002).

### 3.4 Cancer

An estimated 19.3 million people were identified with cancer worldwide in 2020 (18.1 million if nonmelanoma skin cancer is excepted) and about 10 million people lost their lives to the disease. With an expected 2.3 million new cases (11.7%), female breast cancer has exceeded lung cancer as the most frequently diagnosed cancer. This is preceded by lung (11.4%), colorectal (10%), prostate (7.3%), and stomach (5.6%) cancers (Sung et al., 2021). Starch resistant to digestion in the small intestine has a beneficial effect on the colonic luminal environment and protects against colorectal cancer (Le Leu et al., 2007). The antigenotoxic, antimutagenic, and anticarcinogenic characteristics of resistant starch derived from green banana flour were assessed. It is hypothesized that resistant starch reduces the incidence of cancer biomarkers and improves illness outcome by reducing the amount of biomarkers that develop into tumors during carcinogenesis. Therefore, the therapeutic virtues of resistant starch might be used in human dietary applications (Navarro et al., 2015). According to studies, genotoxicity, mutagenicity, and carcinogenicity are aligned exercises in which a numbers of chemical carcinogens can engage with genetic material to make cancer causing mutations (Mauro et al., 2013). Due to the elevated potentiality antigenotoxic, antimutagenic, and anticarcinogenic effects of these substances, studies show diets including prebiotics such as resistant starch have a substantial role in inhibiting colorectal cancer (Clark et al., 2012). Analyzed the effect of a diet great in resistant starch on the expression profile of miRNAs and their target genes, as well as biological procedures and pathways that engage as central character in pancreatic tumors of xenografted mice. Mice given the RS diet had a significantly different expression profile for 19 miRNAs in tumor tissues compared to control mice. These findings advance our thoughtful of the potential role of resistant starch in the development of cancer and suggest an integrative approach for enhancing the usefulness of on‐the‐shelf cancer treatments (Trivieri et al., 2020).

### 3.5 Obesity

Obesity and the disorders that are linked to it, such as type 2 diabetes, ischemic cardiac disease, persistent renal illness and oncological diseases, including a wide range of cancers, are consistently ranked among the most prevalent and important factors in overall global death rates. 25% of the world's adult peoples is said to be afflicted by it right now because of nonalcoholic fatty liver disease (NAFLD). estimated to affect 25 percent of the global adult population at present, making it one of the most noticeable obesity‐related comorbidity in recent years. Implying, no other factors contribute to the accumulation of fat in the liver, such as alcohol misuse, persistent viral hepatitis, or the use of steatosis‐inducing drugs, this condition is referred to as liver steatosis. Increased adipose tissue size and malfunctioning adipocytes are related with increased triglycerides hydrolysis and decreased insulin sensitivity, both of which contribute to decreased liver fatty acid oxidation and intrahepatic triglyceride buildup in obese individuals. Due to insulin resistance, adipose tissue produces abnormal amounts of cytokines and hormones, which triggers an inflammatory pathway and worsens liver lipotoxic damage. The importance of the gut‐liver‐adipose tissue axis is further highlighted by the fact that these impacts disrupt intestinal microbiota and nutrition metabolism (Fan & Cao, 2013; Romero‐Gómez et al., 2017; Zelber‐Sagi et al., 2017). The SCFA synthesis of elevated fat fed mice was improved by feeding them GB (rich in resistant starch), which also decreased the appearance of genes involved in lipogenesis and increased the appearance of transport 16 proteins involved in lipid excretion. Collectively, these results lend credence to the idea that GB has the ability to ameliorate the elevated fat obesity associated hepatic steatosis and, thus, can be engaged as a method to treat obesity‐related illnesses (Rosado et al., 2020). Unripe banana flour (UBF) cookies were made using a minimal ingredient list to cut down on fat and easily absorbed carbs. A nutritious option for persons with health issues including diabetes and obesity, cookies made with UBF showed essential starch digestive features (Agama‐Acevedo et al., 2012). The use of green banana flour was associated with enhanced fecal cholesterol excretion and weight loss prospects. Unripe banana flour was shown to have promise as a therapy for obesity and lipid excretion. There is a need for more research to confirm the mechanisms response to a rise in plasma triacylglycerol ranks in the animals who were fed the highest quantities of banana flour with 20 percent probably due of the elevated levels of resistant starch in the flour (Escobar et al., 2019).

### 3.6 Heart

Bananas are high in phenolics, flavonoid, and catecholamines, which are believed to lessen the risks of getting cardiac disease and cancer (Li et al., 2020). Elevated fiber diets may also be helpful for fecal bulking, shorter transit time, depressing of postprandial glycemic response, keeping blood cholesterol levels in healthy varieties and lessened the risk of coronary cardiac disease (Segundo et al., 2017). Systemic inflammation and the advent of metabolic and cardiovascular disorders are only a couple of the many detrimental effects of obesity (Swinburn et al., 2019; Trindade et al., 2022).

### 3.7 Prebiotic and probiotic effects of green banana

Probiotics are described as “live microorganisms capable of colonizing the gastrointestinal tract (GIT)” and “conferring benefits to human health” when injected in sufficient quantities. These perks include protection from certain cancers, regulation of the intestines, enhanced digestion, decreased lactose intolerance, fewer negative reactions to antibiotics, and alleviation of IBS symptoms (Fao, 2002; Fontana et al., 2013). Prebiotics, or indigestible dietary items that have a favorable result on the host by selectively boosting the expansion and the activity of restricted bacteria in the colon, are progressively being utilized to help diagnose GI Tract problems (Looijer‐van Langen & Dieleman, 2009; Meyer, 2015). Various prebiotics, such as dietary fiber, can be consumed by certain colonic bacteria to create a diversity of metabolites, including short‐chain fatty acids (SCFAs) namely acetic acid, propionic acid, and butyric acid, lactic acid, and gas (Meyer, 2015). Because it contains resistant starch, green banana flour is a prebiotic food., according to research by Asp and Björck (1992). The physicochemical and sensory qualities of yoghurt were enhanced when it was blended with green banana flour at a concentration of up to 5%. Yoghurt's iron and fiber content went up, and its nutritional value went up with it (Abdalla & Ahmed, 2019). Dual step enzymatic dealing employing amylopullulanase and amyloglucosidase substantially helped in the molecular variation of the starch granule, increasing the flour's resistant starch content and allowing for an in vitro evaluation of the flour's prebiotic impact. The capability of dual enzyme treatment. to back the growth of probiotic microorganisms revealed not only its potential as a prebiotic, but also its potential as a synbiotic. The results of this study may have long‐term implications for the use of RS as a nutritional supplement for people with diabetes (Das et al., 2022a). Dietary fibers acquired from agavins and green banana flour are difficult to digestion yet support healthy fermentation by gut microbes. Mice on a BF diet had a substantial reduction in body weight AV, and BF boosted health after fermentation throughout the colon, suggesting that this mechanism moderates the metabolic impacts of HF diets. Joining multiple DFs that ferment at varying rates along the large intestine eventually results in a synergistic impact via SCFA synthesis, and so supported a more visible prebiotic effect that may give a more potential health benefit to the host (Alvarado‐Jasso et al., 2020). According to another study, the inclusion of Green Banana Pulp to fermented milk enhances the solidity of the probiotic strain *L. paracasei* LBC 81 during storage. The development of *L. paracasei* LBC 81 was encouraged, and its viability was maintained, by the addition of GBP throughout a period of refrigerated temperature. This advantage is compounded by the fact that higher concentrations of GBP have less of an impact on the fermented milks' tone, chroma, and color difference. When contrasted to non‐GBP fermented milk and the other formulations, the sensory analysis of fermented milk enhanced with 6.0% GBP showed greater acceptability (Vogado et al., 2018). Raw BPF greatly increased the development of *L. acidophilus* in vitro, due of existence of resistant starch. Every day, male Wistar rats were given raw BPF, which altered their intestinal ecology by favoring the growth of beneficial species while defeating the growth of pathogens. Prebiotic tests using raw banana pulp flour were found to be effective in both vitro and in vivo (Gajananrao Mahore & Shirolkar, 2018). The use of GBP in yoghurt has the ability to have a prebiotic effect, yet it does so without altering the yoghurt's sensory quality or the proportions of its most significant macronutrients or ash. It is possible that the observed influence on textural profile will be useful in developing novel dairy products. When green banana pulp was added to yoghurt, *L. acidophilus* proliferated after just a period of fermentation lasting only 24 h, whereas *B. bifidum* proliferated after a week in cold storage. Without compromising its physicochemical or olfaction properties, the green banana pulp has been exposed to have prebiotic potential (Costa et al., 2017). The developed approach allows for the synthesis of a high‐quality RS with prebiotic characteristics for use in health‐protective applications. Fermenting RS type III from banana starch makes it contain a lot of butyric acid. Bananas satisfy numerous needs, notably for functional food production. They are a good source of carbohydrates that are easy to digest and also have high potassium and magnesium concentrations (Lehmann et al., 2002). Optimizing the fermentation of milk with *L. plantarum* 112 BG was greatly aided by the addition of the GBF, which may be used instead of plantain flour to provide resistant starch, which could function as a prebiotic. *L. helveticus*‐fermented milk showed strong proteolytic and ACE inhibitory activities, as well as plasmid DNA protection. These findings are significant because they suggest that it may be worthwhile to pursue research into the production of fermented milk that has elevated antioxidant activity and peptides with biological properties in order to bring advantages to consumers (Busanello et al., 2019).

## 4 CONCLUSION

The highest RS concentration can be found in unripe green bananas when compared to other entire, unprocessed meals. beneficial source of fiber, vitamins, minerals, resistant starch (RS), and other nutrients. As it has decreased garbage, it has been helpful. Biowaste from banana plants that is high in antioxidants. Resistant starch made from green bananas has been used to treat gluten‐free products. When it is utilized and put to use, it has a favorable impact on the food industry. traits that are useful The fact that green banana resistant starch can be used in chicken mortadella without changing the flavor of the meal shows how practical and technological the starch's properties are. When utilized to make functional foods that are rich in resistant starch, banana flour, which is prepared from green bananas, has been found to have useful properties like pasting, heat stability, and gelatinization. Crystallinity, foaming, and textural features are just a few examples of how it excels as an useful meal. Finally, from a medical point of view, it has been demonstrated to have a beneficial effect on the gut, cancer, diabetes, obesity, immunology, as a prebiotic, a probiotic function, and in cardiovascular disorders. Preventative health measures based on the manufacture of an RS rich in prebiotics. Further, it tends to favor probiotic strains, which have beneficial effects on health, functioning, and food sector applications.

## AUTHOR CONTRIBUTIONS

**Haroon Munir:** Supervision (equal). **Hamza Alam:** Writing – original draft (equal). **Muhammad Tahir Nadeem:** Supervision (equal). **Riyadh S. Almalki:** Writing – review and editing (equal). **Muhammad Sajid Arshad:** Supervision (supporting). **Hafiz Ansar Rasul Suleria:** Supervision (equal).

## CONFLICT OF INTEREST STATEMENT

The authors declare that they have no conflict of interest with each other.

## Data Availability

This is review article and this statement is not applicable to this article.
